# Deciphering Molecular Mechanisms Involved in Salinity Tolerance in Guar (*Cyamopsis tetragonoloba* (L.) Taub.) Using Transcriptome Analyses

**DOI:** 10.3390/plants11030291

**Published:** 2022-01-22

**Authors:** Biswa R. Acharya, Devinder Sandhu, Christian Dueñas, Jorge F. S. Ferreira, Kulbhushan K. Grover

**Affiliations:** 1 U.S. Salinity Lab (USDA-ARS), 450 W Big Springs Road, Riverside, CA 92507, USA; biswaa@ucr.edu (B.R.A.); Jorge.ferreira@usda.gov (J.F.S.F.); 2 College of Natural and Agricultural Sciences, University of California Riverside, 900 University Avenue, Riverside, CA 92521, USA; cduen002@ucr.edu; 3 Department of Plant and Environmental Sciences, New Mexico State University, Las Cruces, NM 88003, USA

**Keywords:** guar, gene expression, qRT-PCR, RNA-Seq, salt stress, salt tolerance, stress, transcriptome, salinity

## Abstract

Guar is a commercially important legume crop known for guar gum. Guar is tolerant to various abiotic stresses, but the mechanisms involved in its salinity tolerance are not well established. This study aimed to understand molecular mechanisms of salinity tolerance in guar. RNA sequencing (RNA-Seq) was employed to study the leaf and root transcriptomes of salt-tolerant (Matador) and salt-sensitive (PI 340261) guar genotypes under control and salinity. Our analyses identified a total of 296,114 unigenes assembled from 527 million clean reads. Transcriptome analysis revealed that the gene expression differences were more pronounced between salinity treatments than between genotypes. Differentially expressed genes associated with stress-signaling pathways, transporters, chromatin remodeling, microRNA biogenesis, and translational machinery play critical roles in guar salinity tolerance. Genes associated with several transporter families that were differentially expressed during salinity included ABC, MFS, GPH, and P-ATPase. Furthermore, genes encoding transcription factors/regulators belonging to several families, including SNF2, C_2_H_2_, bHLH, C3H, and MYB were differentially expressed in response to salinity. This study revealed the importance of various biological pathways during salinity stress and identified several candidate genes that may be used to develop salt-tolerant guar genotypes that might be suitable for cultivation in marginal soils with moderate to high salinity or using degraded water.

## 1. Introduction

Soil salinization is a serious global problem as saline soil severely impacts crop productivity. It has been predicted that problems associated with soil salinity will increase in many parts of the world due to climate change [1]. The effects of soil salinity are aggravated in plants in response to high temperatures due to evapotranspiration, specifically in semiarid and arid regions. Salinity stress affects plants at molecular, biochemical, physiological, morphological, and productivity levels [2]. Although degraded waters negatively affect the productivity of salt-sensitive crops due to their higher salt concentration, they could be a valuable alternative to freshwater to grow salt-tolerant crops.

High concentrations of NaCl in the soil cause a reduction in water potential that in turn leads to reduced availability of water from the soil to the plant, which ultimately induces osmotic stress in plants [3]. Soil ions such as Na^+^ and Cl^−^ enter plants through outer root cells [4]. Then, these ions can be transported to the root xylem and subsequently from roots to shoots. Increasing ions inside plant cells cause an ionic imbalance that leads to immediate osmotic stress followed by ionic toxicity and the production of reactive oxygen species. A salinity-induced increase in Na^+^ in plant cells inhibits the biosyntheses and activities of various metabolic enzymes, induces stomatal closure, and decreases photosynthesis. To prevent the severity of salinity-induced osmotic stress, plants synthesize various compatible solutes and osmoprotectants (betaine, glycine, inositol, mannitol, trehalose, and polyamines) [2,5,6].

The detailed mechanisms by which plants sense salt are not well understood. In plants, salinity stress activates multiple signaling pathways that collectively provide tolerance to salinity [7]. An extracellular salt sensor, monocation-induced [Ca^2+^]_i_ increases 1 (MOCA1), senses Na^+^ and a few other monovalent cations [8]. Glycosyl inositol phosphorylceramide (GIPC) sphingolipids in the plasma membrane are synthesized by MOCA1. GIPCs can bind monovalent cations, such as Na. It has been proposed that the binding of Na ions to GIPCs causes the depolarization of cell-surface potential that leads to the opening of calcium-influx channels, which increases intracellular Ca^2+^ levels. The salt overly sensitive (SOS) pathway is activated upon the increase in intracellular Ca^2+^ levels. Upon binding with Ca^2+^, SOS3 interacts with SOS2 and activates its kinase domain [9]. In turn, SOS2 phosphorylates SOS1, which transports Na^+^ from inside to outside of the cell [10]. These facts indicate that calcium and SOS signaling pathways play critical roles during salinity stress.

Calcium is a vital secondary messenger in response to salinity stress. The calcium signaling pathway regulates many types of cellular machinery (e.g., transporters) and signaling components (e.g., CIPKs and CBLs) that help in metal uptake regulation and the maintenance of homeostasis of potassium-, magnesium-, and nitrogen-containing homeostasis [11].

Reactive oxygen species (ROS) are secondary messengers in response to various stress signaling pathways, including salinity stress [12]. Excess production of ROS in response to salinity induces oxidative stress that leads to oxidative damage of nucleic acid, proteins, and membrane lipids [13]. Plants also synthesize enzymatic and non-enzymatic antioxidants that protect cellular components and macromolecules from oxidative stress. Enzymatic antioxidants that perform detoxification of ROS, include catalase (CAT), peroxidase (POX), superoxide dismutase (SOD), and enzymes associated with ascorbate (ASC)-–glutathione cycle, ASC peroxidase (APX), glutathione reductase (GR), dehydroascorbate reductase (DHAR), and monodehydroascorbate dehydrogenase (MDHAR) [14,15].

Phytohormone signaling pathways play crucial roles in salinity tolerance in plants by regulating development, growth, and adaptation [16]. During salinity stress, plant stress hormones [abscisic acid (ABA), jasmonic acid (JA), salicylic acid (SA), and ethylene] and plant growth hormones [auxin (IAA), gibberellins (GA), and cytokinin] play modulatory roles (positive and negative) with intricate crosstalk that contribute to plant growth adaptation during salinity stress. In response to salinity, differential expression of genes associated with phytohormone biosynthesis, signaling, and transport mechanisms have been reported in different plant species, which could be important determinants for salinity tolerance [17,18,19].

Membrane transport mechanisms play a vital role in salinity tolerance in plants [20]. Several transporters have been identified in different plants linked to salinity tolerance. For example, proton pumps transport solutes and ions across membranes. Non-selective cation channels are permeable to many monovalent cations, which have been suggested to play roles in an influx of Na^+^ across the plasma membrane in root cortical cells [21,22]. Hence, the differential expression of various transporters linked to salinity tolerance is critical, primarily regulated by a specific set of transcription factors.

Transcription factors are key regulators of gene expression in response to various stresses, including salinity stress [23]. Transcription factors regulate the expression of genes that are required for salt sensing, signal transduction, transport, ion homeostasis, and various other genes involved in salinity tolerance [24].

Guar (*Cyamopsis tetragonoloba* (L.) Taub.) is a self-pollinated legume crop mostly grown in resource-deficient conditions in semiarid and arid regions because of its drought and heat tolerance [25,26]. About 80% of the world guar is produced in Rajasthan, India, as a rain-fed crop from July to early November. The rest is produced the USA, Brazil, South Africa, Malawi, Zaire, Sudan, Australia, and China, with 38% of Indian guar being exported to the US in 2014–2016 (https://agriculture.rajasthan.gov.in/content/dam/agriculture/Rajasthan%20Agricultural%20Competitiveness%20Project/valuechainreport/RACP_VC_Guar.pdf; accessed on 13 January 2022). Recent research efforts focus on the production of guar in the southwest US, which is often affected by high water and soil salinity [27,28,29].

The endosperm of guar seeds is a rich source of a natural hydrocolloid (galactomannan), popularly known as guar gum. Guar gum is used in various industries, including food, textile, petroleum, natural gas, mining, pharmaceutical, cosmetics, paper, and paint, making guar an important industrial crop [30]. The productivity of guar is negatively affected by salinity [31,32,33]. Hence, identification and molecular analysis of salt-tolerant genotypes are essential for guar productivity under saline conditions. Transcriptome analysis, combined with phenotypic and physiological characterizations of salt-tolerant vs. salt-sensitive genotypes, will further unveil the importance of different pathways that are critical for salinity tolerance in guar. This understanding may become instrumental in developing new salt-tolerant guar genotypes that could be grown under saline conditions without a significant crop productivity compromise. Successful guar cultivation in the U.S. will reduce the burden of importing guar gum from foreign countries and increase farmers’ profitability by efficiently utilizing marginal soils and recycled water for crop cultivation.

A few preliminary studies have been performed to examine the salinity tolerance of different guar genotypes [32,33,34,35,36,37]. Salinity stress affects guar development, growth, and productivity. Guar seed yield was reduced in response to irrigation-water salinity above 8.8 dS m^−1^, whereas vegetative growth was reduced above 4.9 dS m^−1^, with its root system adapted to salinity tolerance [38,39].

Matador is an improved guar cultivar with higher yields than other U.S. guar cultivars due to its prolific branching [27]. A recent field study indicated that the genotype PI 340261 had similar traits as Matador, except for branching [31]. In a previous study, ‘Matador’ was classified as salt-tolerant and ‘PI 340261’ as salt-sensitive, based on salt tolerance index (STI) for root length, shoot length, root biomass, shoot biomass, and their tissue Na, and Cl accumulation [31]. This study applied the RNA-Seq approach to study molecular mechanisms of salinity tolerance and salinity-regulated transcriptomes in leaves and roots of the salt-tolerant ‘Matador’ (genotype ‘3’) and the salt-sensitive ‘PI 340261’ (genotype ‘22’) under control and salinity conditions.

## 2. Results

### 2.1. De Novo Transcript Assembly and Functional Annotation

To understand the molecular mechanism of salinity tolerance of guar at the transcriptome level of the salt-tolerant ‘Matador’ (‘03’, hereafter) and salt-sensitive ‘PI 340261’ (‘22’, hereafter), a three-factor RNA-Seq experiment was performed with two levels/factors to examine differential gene expression between variables: genotypes (03 and 22), tissue types (root and leaf), and treatment types (control and salt treatment). The experimental samples were named as C03L for Control ‘03’ Leaf, C22L for Control ‘22’ Leaf, T03L for Treatment ‘03’ Leaf, T22L for Treatment ‘22’ Leaf, C03R for Control ‘03’ Root, C22R for Control ‘22’ Root, T03R for Treatment ‘03’ Root, and T22R for Treatment ‘22’ R (Table S1). Our analyses identified a total of 527,407,683 clean reads, consisting of 158 gigabases (Gb) with an average of 6.6 Gb/library (Table S2). A total of 296,167 transcripts (≥200 bp) were identified, with transcript sizes ranging from 200 bp to 19,633 bp, of which 46% had more than 1000 bp. Our data analysis identified a total of 296,114 unigenes, which ranged from 301 bp to 19,363 bp with a mean length of 1494 bp (Figure S1).

To obtain broad functional annotation of all unigenes, seven databases were searched, which revealed substantial sequence similarity in the databases ranging from 90,848 unigenes (30.68%) in KO (KEGG ortholog) to 201,592 unigenes (68%) in NR (NCBI non-redundant protein sequences) databases. In addition, our analyses indicated that 228,611 unigenes (77%) were annotated in at least one database, whereas 19,214 (6.4%) unigenes were found to be annotated in all seven databases (Figure S2).

### 2.2. Differentially Expressed Genes (DEGs) in Response to Salt Stress

**Figure 1:**
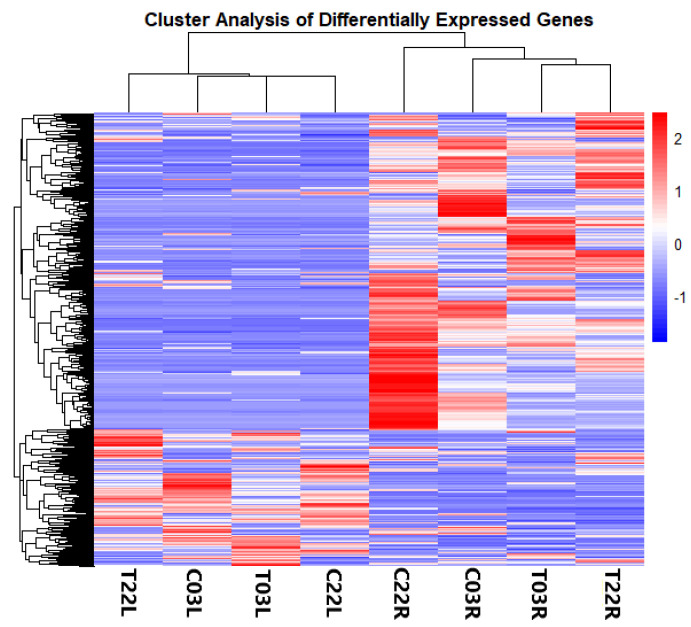
Heatmap-based clustering of differentially expressed genes (DEGs). The heatmap and hierarchical clustering show the status of gene expression and clustering of DEGs (those that were differentially expressed at least in one comparison) across all eight indicated samples in a specific column.

To analyze differential gene expression, gene expression levels of all 24 libraries were calculated by mapping reads of each replicate compared to the assembled transcriptome. Of all the reads, 93% were mapped to the assembly (Table S2) with 65,703 DEGs, and 22% of the total identified clusters (296,114) being present at least in one of the comparisons: treatment vs. control, ‘03’ (salt-tolerant) vs. ‘22’ (salt-sensitive), or leaf vs. root (Figure 1 and Supplemental Table S3). The cluster analysis based on gene expression indicated two major groups according to tissue types: root and leaf (Figure 1). Genes of T03R and C03R formed the first subgroup, whereas T22R and C22R formed the second subgroup within the root group. In the leaf group, subgroupings were also based on genotypes, as observed in the root group (Figure 1).

DEGs were identified in treatment vs. control, ‘03’ (salt-tolerant) vs. ‘22’ (salt-sensitive), and leaf vs. root comparisons by gene expression analyses (Figure S3 and Table 1). In treatment vs. control comparisons, 853 (357 upregulated and 496 downregulated), 8283 (1805 upregulated and 6478 downregulated), 1070 (644 upregulated and 426 downregulated), 1006 (511 upregulated and 495 downregulated) DEGs were identified when comparing T03L vs. C03L, T03R vs. C03R, T22L vs. C22L, and T22R vs. C22R, respectively. In salt-tolerant (‘03’) vs. salt-sensitive (‘22’) comparisons, 1049, 1355, 1031, and 1727 DEGs were identified in C03L vs. C22L, C03R vs. C22R, T03L vs. T22L, and T03R vs. T22R, respectively. In leaf vs. root comparisons, 41,540, 35,936, 21,383, and 27,383 DEGs were identified in C03L vs. C03R, C22L vs. C22R, T03L vs. T03R, and T22L vs. T22R, respectively (Table 1, Figure S3, and Table S3).

### 2.3. Verification of DEGs Using Quantitative Reverse Transcription PCR (qRT-PCR)

**Figure 2:**
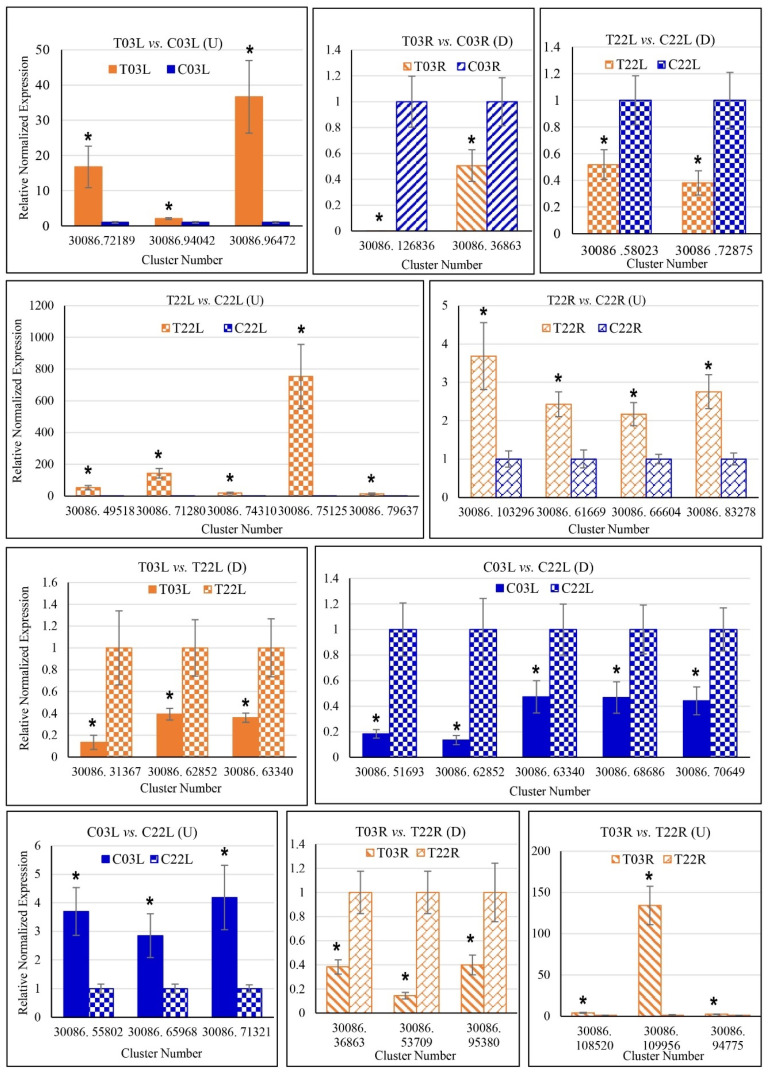
qRT-PCR validation of gene expression observed through RNA-Seq analysis. The *y*-axis indicates relative normalized expression, and the *x*-axis indicates cluster number (gene IDs). Graphs for upregulated (U) and downregulated (D) genes are shown separately. An asterisk (*) indicates a significant difference (t-test at *p* ≤ 0.05). T03L, Treatment ‘03’ Leaf; C03L, Control ‘03’ Leaf; T22L, Treatment ‘22’ Leaf; C22L, Control ‘22’ Leaf; T22R, Treatment ‘22’ Root; C22R, Control ‘22’ Root, T03R, treatment ‘03’ Root, and C03R, Control ‘03’ Root.

RNA-Seq data validation was performed employing the qRT-PCR assay for 33 randomly selected guar gene clusters for different comparisons (Tables S4 and S5). Out of 33 gene clusters, 27 were studied for single comparisons, and three were evaluated for two different comparisons (Figure 2). Comparisons were performed for relative normalized expressions data for all tested genes (clusters) (Figure 2). The general trend of differential expression profiles observed by both qRT-PCR and RNA-Seq methods was similar for most genes (clusters), indicating the validity of the RNA-Seq results.

### 2.4. Gene Ontology (GO) Enrichment Analysis of DEGs

To examine significant functional enrichment of DEGs in treatment vs. control and salt-tolerant (‘03’) vs. salt-sensitive (‘22’) genotypes, GO enrichment analyses were performed for three main categories: biological process (BP), molecular function (MF), and cellular components (CC) (Table S6). In treatment vs. control comparisons, 3, 2, 6, and 7 GO terms were significantly enriched in T03L vs. C03L, T03R vs. C03R, T22L vs. C22L, and T22R vs. C22R, respectively (Table S6). Among these, GTPase activity (GO:0003924) was significantly enriched in the T03R vs. C03R. In salt-tolerant (‘03’) vs. salt-sensitive (‘22’) comparisons, two GO terms, including transmembrane transport (GO:0005794), were significantly enriched in T03L vs. T22L (Table S6).

### 2.5. KEGG Enrichment Analysis of DEGs

KEGG enrichment analyses of DEGs were performed in all pairwise comparisons to identify enriched biological pathways (Supplemental Table S7). In salt treatment vs. control comparisons, 6, 43, 0, and 45 pathways were significantly enriched in T03L vs. C03L, T03R vs. C03R, T22L vs. C22L, and T22R vs. C22R, respectively. Among these, MAPK signaling pathway (ko04011) was enriched in T03L vs. C03L, plant hormone signal transduction (ko04075), and glutathione metabolism (ko00480) pathways were enriched in T03R vs. COR, and brassinosteroid biosynthesis pathway (ko00905) was enriched in T22R vs. C22R. In salt-tolerant (03) vs. salt-sensitive (22) comparisons, 0, 40, 4, and 6 pathways were significantly enriched in C03L vs. C22L, C03R vs. C22R, T03L vs. T22L, and T03R vs. T22R, respectively. Among these pathways, zeatin biosynthesis (ko00908) was enriched in C03R vs. C22R and sphingolipid metabolism (ko00600) in T03L vs. T22L (Table S7).

### 2.6. DEGs Associated with Stress Pathways

**Figure 3:**
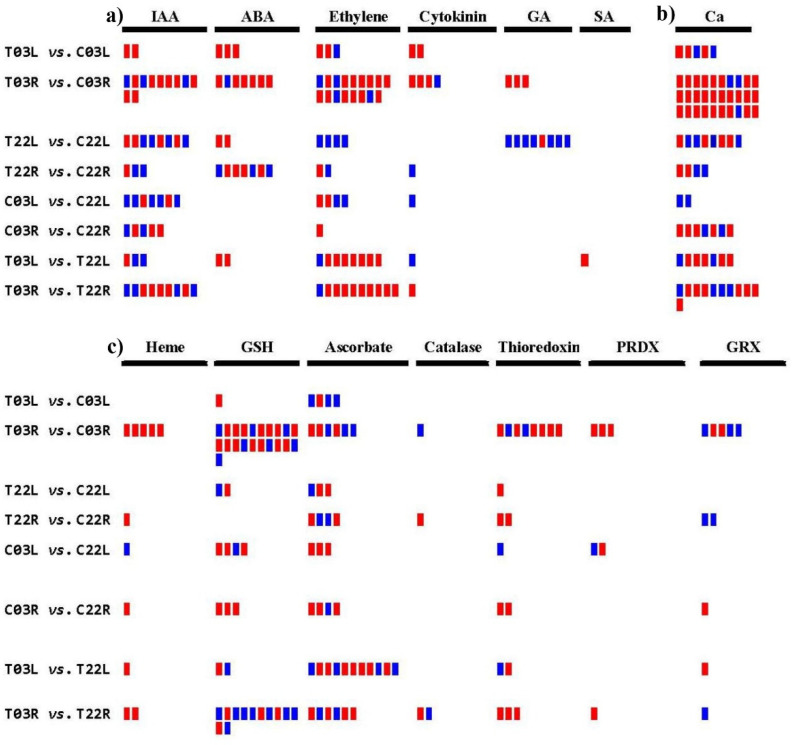
DEGs associated with phytohormone, calcium signaling, and redox pathways. (**a**) DEGs associated with phytohormone pathways (are indicated at the top). (**b**) DEGs associated with calcium signaling (Ca). (**c**) DEGs associated with redox signaling pathways (indicated at the top). The red color indicates downregulated DEGs, and the blue indicates upregulated DEGs. Left-side texts show pairwise comparisons. The top four compared samples indicate treatment vs. control, and the bottom four compared samples indicate salt-sensitive vs. salt-tolerant comparisons. IAA, indole acetic acid (auxin); ABA, abscisic acid; GA, gibberellins; SA, salicylic acid; Ca, calcium; GSH, glutathione; PRDX, peroxiredoxin; GRX, glutaredoxin.

In plants, various pathways contribute to salinity stress signaling. All DEGs were examined to find out their association with phytohormone signaling, calcium signaling, and redox signaling, as these pathways are known to play critical roles in response to salinity stress (Figure 3 and Tables S8–S10).

#### 2.6.1. Hormonal Signaling

In treatment vs. control comparisons, 10 DEGs were identified in T03L vs. C03L, two downregulated for IAA, three downregulated for ABA, two downregulated and one upregulated for ethylene, and two downregulated DEGs for cytokinin. In T03R vs. C03R, 11 DEGs were found for IAA (eight downregulated and three upregulated), seven were for ABA (six downregulated and one upregulated), 17 were for ethylene (14 downregulated and 3 upregulated), four were for cytokinin (three downregulated and one upregulated), and three downregulated DEGs were for GA. In T22L vs. C22L, eight DEGs were detected for IAA (four downregulated and four upregulated), two downregulated DEGs for ABA, four upregulated DEGs for ethylene, and eight for GA (one downregulated and seven upregulated). In T22R vs. C22R, 3 DEGs were identified for IAA (one downregulated and two upregulated), seven were for ABA (four downregulated and three upregulated), two were for ethylene (one downregulated and one upregulated), and one upregulated DEG was for cytokinin. When comparing salt-tolerant vs. salt-sensitive groups, 12, 6, 15, and 20 DEGs associated with hormonal signaling were identified in C03L vs. C22L, C03R vs. C22R, T03L vs. T22L, T03R vs. T22R, respectively. We observed that the highest number of DEGs was associated with ethylene signaling, followed by IAA and ABA (Figure 3a and Table S8).

#### 2.6.2. Calcium Signaling

In treatment vs. control comparisons, 5, 30, 8, and 4 DEGs associated with calcium signaling were observed in T03L vs. C03L, T03R vs. C03R, T22L vs. C22L, and T22R vs. C22R, respectively. In salt-tolerant vs. salt-sensitive comparisons, 2, 7, 7, and 11 DEGs related to calcium signaling were identified in C03L vs. C22L, C03R vs. C22R, T03L vs. T22L, and T03R vs. T22R, respectively (Figure 3b and Table S9).

#### 2.6.3. Redox Signaling

In treatment vs. control comparisons, 5, 49, 6, and 10 DEGs associated with redox signaling were identified in T03L vs. C03L, T03R vs. C03R, T22L vs. C22L, and T22R vs. C22R, respectively. In salt-tolerant vs. salt-sensitive comparisons, 11, 11, 17, and 27 DEGs related to redox signaling were identified in C03L vs. C22L, C03R vs. C22R, T03L vs. T22L, and T03R vs. T22R, respectively. Our analysis revealed the highest number of DEGs was associated with GSH, followed by ascorbate and thioredoxin (Figure 3c and Table S10).

### 2.7. DEGs Associated with Transporters

**Figure 4:**
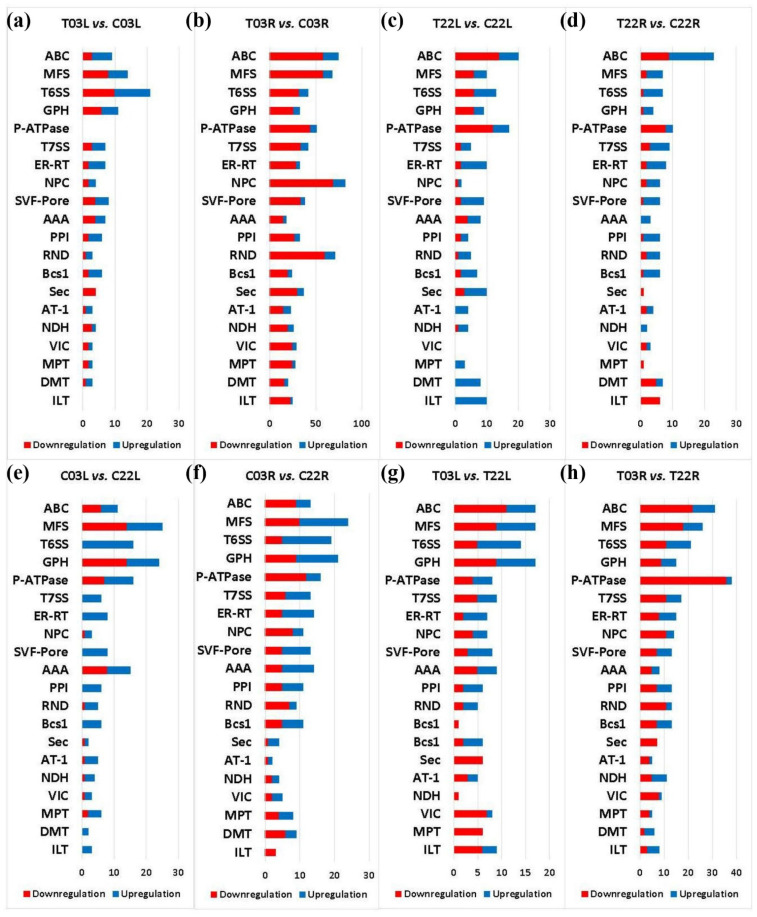
Transporter analysis of DEGs. The *y*-axis indicates transporter superfamilies. The *x*-axis shows gene counts. Salt treatment vs. control comparisons are shown in the top four panels (**a**–**d**). Salt-tolerant vs. salt-sensitive comparisons are shown in the bottom panels (**e**–**h**). The red color indicates downregulated DEGs, and the blue indicates upregulated DEGs. Compared samples are indicated at the top of each panel.

Transporters are vital for ion distribution and homeostasis throughout the plants. A previous study indicated that in response to salinity stress, guar genotype 03 (Matador) showed a lower accumulation of Na and other ions in comparison to a salt-sensitive genotype 22 (PI 340261), which encouraged us to identify genes encoding transporters [31]. We identified a total of 2764 DEGs encoding transporters in treatment vs. control comparisons and salt-tolerant vs. salt-sensitive comparisons (Figure 4 and Tables S11 and S12).

In treatment vs. control comparisons, 1614 DEGs encoding various families of transporters were identified. In T03L vs. C03L, 48 families of transporters encoded by 168 DEGs were identified, including the monovalent cation: proton antiporter-1 (CPA1) family. In T03R vs. C03R, 1114 DEGs encoding 87 transporter families, including the K^+^ uptake permease (KUP) family, were found. In T22L vs. C22L, 228 DEGs encoding 56 transporter families were detected, which included the auxin efflux carrier (AEC) family and the voltage-gated ion channel. In T22R vs. C22 R, 184 DEGs encoding 58 transporter families were found, including the monovalent cation (K^+^ or Na^+^): proton antiporter-3 (CPA3) family (Tables S11 and S12).

When comparing salt-tolerant vs. salt-sensitive guar cultivars, 1150 DEGs encoding numerous families of transporters were identified. In C03L vs. C22L, 239 DEGs encoding 52 transporter families were detected, including the voltage-gated ion channel (VIC) superfamily and the P-type ATPase superfamily. In C03R vs. C22R, 286 DEGs encoding 55 transporter families were found, including the major intrinsic protein (MIP) family. In T03L vs. T22L, 226 DEGs encoding 47 transporter families were identified, including the ATP-binding cassette (ABC) superfamily. In T03R vs. T22R, 399 DEGs encoding 59 transporter families were recognized, including the ankyrin family transporters (Tables S11 and S12).

### 2.8. DEGs Associated with Transcription Factors/Regulators

**Figure 5:**
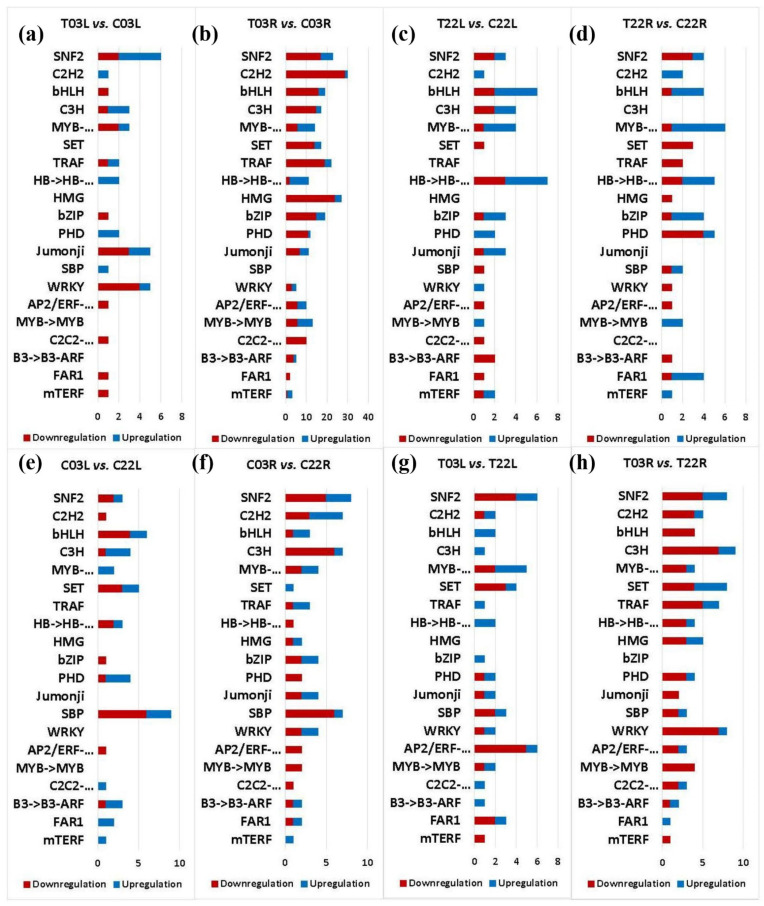
Transcription factor analysis of DEGs. The *y*-axis indicates transcription factor families. The *x*-axis shows gene counts. Salt treatment vs. control comparisons are shown in the top four panels (**a**–**d**). Salt-tolerant vs. salt-sensitive comparisons are shown in the bottom panels (**e**–**h**). The red color indicates downregulated DEGs, and the blue indicates upregulated DEGs. Compared samples are indicated at the top of each panel.

Transcription factors are major regulators of gene expressions during growth, development, and in response to various stresses, including salinity. Thus, we focused on identifying DEGs encoding transcription factors in salt-tolerant ‘03’ and salt-sensitive ‘22’ guar genotypes in response to salinity stress. Our analyses revealed a total of 962 transcription factors/regulators in treatment vs. control and salt-tolerant vs. salt-sensitive comparisons (Tables S13 and S14). In treatment vs. control companions, 69, 394, 69, and 68 transcription factors/regulators were identified in T03L vs. C03L, T03R vs. C03R, T22L vs. C22L, and T22R vs. C22R, respectively. In salt-tolerant vs. salt-sensitive comparisons, 74, 96, 77, and 115 transcription factors/regulators were identified in C03L vs. C22L, C03R vs. C22R, T03L vs. T22L, and T03R vs. T22R, respectively (Tables S13 and S14). Differential expressions of genes encoding transcription factors such as MYB in C03L vs. C22L, GRAS in C03R vs. C22R, bHLH in T03L vs. T22L, and GRAS in T03R vs. C03R were observed (Figure 5 and Table S13).

## 3. Discussion

RNA-Seq is one of the most suitable approaches to study gene expression at the transcriptome level that enables researchers to discover genes that play critical roles in specific tissues, physiological conditions, and metabolic pathways in response to various stresses [26,40,41,42,43,44,45,46,47]. The primary goal of this work was to find molecular mechanisms of salinity tolerance in guar. To achieve this goal, we performed RNA-Seq experiments, using the salt-tolerant guar genotype Matador (‘03’) and the salt-sensitive genotype PI 340261 (‘22’), both previously confirmed for their respective salt tolerance and sensitivity [31].

Comparative transcriptome analyses were performed between ‘03’ and ‘22’ in root and leaf of one-month-old guar plants in response to control irrigation water and saline water with electrical conductivity (EC_iw_) of 13.65 dS m^−1^ (Table 2). As the guar genome has not been sequenced, guar de novo assembly was constructed from all sequencing libraries. Our analyses revealed a total of 296,114 non-redundant guar unigenes. A few guar transcriptomics studies have been reported previously. RNA-Seq studies of two guar varieties (M-83 and RGC-1066) had reported the identification of 62,146 unigenes for the leaf tissue [40] and 102,479 unigenes for the root tissue [43]. Other guar studies focusing under different environmental conditions revealed 48,007 to 85,395 unigenes [41,44,46]. Variation in the number of unigenes observed in different reports could be due to multiple factors including the number of genotypes used, number of tissues used, number of treatments used, developmental stage of the tissues used, the quality of RNA, the quality of RNA-Seq data, and the stringency used for data analyses.

Functional annotation analyses of our data showed that 77% of all identified unigenes were protein-coding genes, as they were found in at least one database out of the seven public databases (Figure S2). The remaining 23% of unigenes may not have known protein domains and/or some of these could be non-coding RNA genes.

Differential gene expression analyses revealed a higher number of DEGs in treatment vs. control comparisons than salt-tolerant vs. salt-sensitive comparisons, suggesting that expression differences between salt treatment and control were more pronounced than between the two genotypes. In treatment vs. control comparisons, the highest number of DEGs was observed in the T03R vs. C03R (Table 1). Eight times higher DEGs observed in T03R vs. C03R than T22R vs. C22R indicated that the root of ‘03’ was more responsive to salinity than the root of ‘22’, resulting in better salinity tolerance of ‘03’. Our analyses revealed that a wide variety of genes contributed toward the salinity tolerance of ‘03’ compared to ‘22’, including negative regulators of the SOS signaling pathway, negative regulators of oxidative stress, metabolic enzymes, pathogen defense signaling, hormonal signaling, calcium signaling, redox signaling, transporters, transcription factors, chromatin remodeling factors, microRNA biogenesis, and translational machinery.

Phytohormones play key roles during salinity stress. A maximum number of DEGs for ethylene signaling followed by IAA and ABA signified the higher importance of these three phytohormones in response to salt stress in guar. More DEGs linked with plant hormone signaling were observed in treatment vs. control comparisons than salt-tolerant vs. salt-sensitive comparisons. In treatment vs. control comparisons, a higher number of DEGs associated with phytohormone was observed in roots than leaves in ‘03’. In contrast, we observed more DEGs associated with phytohormone in leaves than roots in ‘22’. These findings indicate that the contrasting tissue-specific expression of genes associated with phytohormone signaling may be one of the contributing factors for differential salinity tolerance abilities of 03 and 22 guar genotypes (Table S8).

A higher number of DEGs associated with calcium signaling was observed in treatment vs. control comparisons than salt-tolerant vs. salt-sensitive comparisons, indicating that induction or suppression of genes involved in calcium signaling is critical during salinity stress in guar (Figure 3). A higher expression level of a gene encoding calcium-dependent protein kinase 26 (CPK26) was observed in T03L compared to C03L and in T03R compared to C03R (Table S9). Overexpression of *Vitis amurensis CPK26* or *Stipa purpurea CIPK26* in Arabidopsis provided tolerance to salinity [48,49]. A higher expression of a gene encoding *CIPK26* in treatment than control in the leaves and roots of ‘03’ demonstrate its positive regulatory role in salinity tolerance in the genotype 03 (Table S3).

Our redox signaling pathway data identified the highest number of DEGs linked with glutathione followed by ascorbate, suggesting the importance of these genes in redox signaling in response to salinity stress (Figure 3 and Table S10). Additionally, DEGs encoding/associated with catalase were observed, suggesting their roles in ROS homeostasis in guar in response to salinity stress. It is known that E3 ubiquitin ligase PQT3 (PARAQUAT TOLERANCE 3) is a negative regulator of oxidative stress. The loss of function or downregulation of this gene protects plants in response to salinity stress by negatively affecting the severity of salinity-induced oxidative stress that protects plants in response to salinity stress [50]. We observed drastic downregulation of a gene encoding E3 ubiquitin ligase PQT3 (cluster-30086.17545) in T03L compared to C03L, but no differential expression was observed in T22L vs. C22L (Table S3). Our findings suggest that the downregulation of guar *E3 ubiquitin ligase PQT3* in genotype ‘03’ may have contributed to higher salinity tolerance in ‘03’ compared to ‘22’.

The homeostasis of ions including Na^+^ and K^+^ is critical for salinity tolerance in plants. In response to salinity, guar genotype 03 accumulates less Na in leaves than genotype 22 [31]. A higher number of DEGs encoding unique transporter families was observed in treatment vs. control comparisons than salt-tolerant vs. salt-sensitive comparisons, indicating inducible/repressible expression of various transporters in response to salinity stress in guar (Table S12).

CPA1 (cation/proton antiporter gene 1) has been implicated in the positive regulation of salinity tolerance [51]. We observed upregulation of various genes encoding CPA1 family in ‘03’ compared to ‘22’ in different comparisons: cluster-30086.145590 in T03L vs. C03L, cluster-30086.113349 in T03R vs. C03R, cluster-30086.145593 in C03L vs. C22L, cluster-30086.82459 in C03R vs. C22R, and cluster-30086.145593 in T03 L vs. T22L (Table S11). Additionally, it is also known that guar *SOS1* (*NHX7*) is upregulated in response to salinity [31]. Our findings suggest that genes encoding CPA family members may have contributed to better salinity tolerance in ‘03’ compared to ‘22’. GIGANTEA is a flowering time controller that negatively regulates salinity tolerance by inhibiting SOS2 protein kinase, an essential component of the SOS-signaling pathway [52]. We observed downregulation of a gene encoding GIGANTEA (cluster-30086.81200) in T03L vs. C03L but not in T22L vs. C22L (Table S3). The downregulation of poplar *GIGANTEA*-like gene expression has been shown to improve salinity tolerance and enhance biomass under salinity [53]. Our findings suggest that the downregulation of a gene encoding GIGANTEA in genotype 03 may have increased salinity tolerance and biomass in salinity conditions.

Potassium homeostasis is necessary during salinity stress indicating that potassium is a determinant factor for salinity tolerance [54]. Genotype 03 (Matador) showed higher K accumulation than genotype 22 (PI 340261) in root tissues [31]. Our transporter analysis revealed a higher expression of a gene encoding a potassium uptake permease (KUP) family protein (cluster-30086.79861) in T03R compared to C03R. In Arabidopsis, KUP family proteins (also known as HAK/KT) play roles in K translocation and transport [55]. Therefore, the gene (cluster-30086.79861) encoding KUP may have contributed to a higher accumulation of K in ‘03’ in response to salinity stress. Higher expression of a gene encoding SKD1 (protein suppressor of K^+^ transport growth defect 1) in T03L compared to C03L indicates that SKD1 contributes to salinity tolerance in the guar genotype 03 [56].

Differential expressions of various genes encoding transporters in treatment vs. control and salt-tolerant vs. salt-sensitive comparisons may have resulted in differential growth responses in guar. Genes encoding important transporters showed differential expression between salinity treatment vs. control or/and salt-tolerant vs. salt-sensitive comparisons, which included genes encoding ABC transporter family, MFS, T6SS, GPH, and P-ATPase (Figure 4). Our analysis indicated upregulation of a gene (cluster-30086.87927) in T03R compared to C03R (Table S11). The gene (cluster-30086.87927) encoding an ABC transporter C family member may have contributed toward salinity tolerance in ‘03’. The overexpression of an ABC transporter has been shown to provide tolerance to salinity stress [57].

Genes encoding GPH and P-ATPase showed a higher level of differential expressions in salt-tolerant vs. salt-sensitive comparisons compared to treatment vs. control comparisons, suggesting that these transporters may be critical for genotype-specific differences. We observed upregulation of a gene (cluster-30086.114412) encoding an MFS transporter in T03R compared to T22R. Several transporters that belong to the major facilitator superfamily (MFS) are known to play positive roles in salinity tolerance, which suggests that genes encoding MFS transporters may have contributed toward salinity tolerance in guar [58]. This study identified upregulation of a gene (cluster-30086.54118) encoding for a GPH (glycoside–pentoside–hexuronide) transporter in T03L compared to C03L. The GPH transporter is known as a sucrose:H^+^ symporter in plants [59,60]. In Arabidopsis, sucrose transporters play positive regulatory roles in salinity tolerance [61]. These facts suggest that genes encoding GPH/sucrose transporters play positive roles and provide tolerance to salinity in guar. The upregulation of a gene encoding P-ATPase (cluster-30086.493) was observed in T03L compared to T22L (Table S11). P-ATPase facilitates Na^+^ extrusion in response to salinity which in turn helps maintain ion homeostasis [62]. These examples suggest that various transporters play critical roles in salinity tolerance in guar.

Various transcription factors regulate gene expression in response to salinity stress. Additionally, several proteins modulate transcriptional processes and are known as transcriptional regulators. For instance, multiple proteins (chromatin remodelers) are associated with chromatin remodeling processes that aid in converting the transcriptionally inactive state of chromatin to active. The most prominent genes encoding transcription factors in our study included C_2_H_2_, bHLH, C3H, and MYB. We identified the upregulation of a gene (cluster-30086.79202) encoding for the C_2_H_2_-type zinc finger protein in T03L compared to C03L in response salinity (Figure 5 and Table S13). Higher expression of C_2_H_2_-type zinc finger proteins has been shown to improve salinity tolerance in plants, indicating that genes encoding C_2_H_2_-type zinc finger proteins may have contributed to better salinity tolerance in genotype 03 compared to 22 [63]. Higher expression of a gene (cluster-30086.34912) encoding a bHLH transcription factor was observed in T03L compared to T22L (Figure 5 and Table S13). Additionally, the higher expression of a gene encoding transcription factor bHLH112 that was observed in T03L compared to C03L suggests its positive role in salinity tolerance [64]. The positive regulatory roles of bHLH transcription factors have been shown in different plants [65]. Our data analysis revealed higher expression of a gene encoding zinc finger C3H domain-containing protein 53 (cluster-30086.92972) in T03R than T22R (Figure 5 and Table S13), suggesting that this gene might be a contributor to salinity tolerance in guar [66]. We observed upregulation of a gene (cluster-30086.34639) encoding an MYB transcription factor in T03R compared to C03R in response to salinity (Figure 5 and Table S13). It is known that MYB transcription factors contribute to salinity tolerance in plants [67]. Transcriptional regulators play essential roles in transcriptional reprogramming during development, physiological and stress responses. The SNF2 protein is a critical component of the chromatin remodeling complex [68]. The involvement of SNF2 has been shown in response to abiotic stress. Our data revealed upregulation of a gene (cluster-30086.108041) encoding SNF2 in T03L compared to C03L, indicating a possible regulatory role of SNF2 in providing salinity tolerance in guar mediated by chromatin dynamics (Figure 5 and Table S13). Chromatin remodeling factors alter the structure of chromatin and are vital regulators of eukaryotic gene expression. The Arabidopsis chromatin remodeling factors have been shown to play regulatory roles in response to biotic and abiotic stresses in plants [69,70]. Our data showed higher expression of chromatin remodeling 5-like (cluster-30086.80282) in T03R than T22R, suggesting its role in regulating gene expression in response to salinity (Table S3). Our findings indicate both transcription factors and transcription regulators play critical roles in providing salinity tolerance in guar.

MicroRNAs (miRNA) are a class of non-coding RNA that play critical roles in regulating the expression of genes at the post-transcriptional level, which is important for plant growth, development, and adaptation to various biotic and abiotic stresses, including salinity stress [71]. Our data also showed higher expression of a gene encoding serine-/arginine-rich splicing factor RS41-like isoform X2 (cluster-30086.82952) in T03R than T22R. It has been reported that, in addition to pre-mRNA splicing, RS41 is also involved in miRNA biogenesis along with RNA binding protein HOS5 and serine-/arginine-rich splicing factor RS40 [72]. Our findings suggest that higher expression of serine-/arginine-rich splicing factor RS41-like isoform X2 gene (cluster-30086.82952) in ‘03’ may contribute to salinity tolerance via miRNA-mediated regulation in response to salinity in guar.

The differential expression of genes associated with translational machinery has been shown to be involved in salinity tolerance [73]. For example, in Arabidopsis, elongation factor 1-alpha is a positive regulator in response to salinity stress [73]. Our RNA-Seq data showed higher expression of a gene encoding for an elongation factor 1-alpha (cluster-30086.137964) in T03L than T22L, indicating that guar elongation factor 1-alpha may be a contributor for salinity tolerance in guar genotype 03 (Table S3).

## 4. Materials and Methods

### 4.1. Plant Material and Salt Treatment

Seeds of a salt-tolerant genotype 03 (Matador, PI 28699) and a salt-sensitive genotype 22 (PI 340261) were sown in lysimeters at the USDA ARS greenhouse (33.973265 latitude, −117.321158 longitude). Plants were watered two times a day with modified half Hoagland’s solution for three weeks to provide macronutrients. Then, plants were treated with saline water, while modified half Hoagland’s solution was used as a control (Table 2). The salt concentration was increased incrementally in four days to avoid osmotic shock. Root and leaf samples were collected for RNA isolation after 48 h of full salinity treatment.

### 4.2. RNA Extraction and Transcript Sequencing

Total RNA isolation from root and leaf tissues was performed using TRIzol reagent (Invitrogen, Carlsbad, CA, USA). Nanodrop was used for the preliminary quantitation of total RNA. Subsequently, Agarose gel electrophoresis was performed to examine RNA degradation and any potential contamination. The 2100 Bioanalyzer (Agilent Technologies, Santa Clara, CA, USA) was used to check the integrity and quantitation of all total RNA samples. Poly-T oligo-attached magnetic beads were used to purify mRNA from total RNA. The mRNA samples fragmented using fragmentation buffer were used for first-strand cDNA synthesis using reverse transcriptase and random hexamers. The product of first-strand synthesis (the cDNA-mRNA hybrid) was used as the template for a nick translation reaction [74]. The second-strand synthesis buffer (Illumina, San Diego, CA, USA), dNTPs, RNase H, and *E. coli* polymerase I were used to generate the second strand. AMPure XP beads were used to purify the cDNA. Then, A-tailing, ligation of sequencing adapters, size selection, and PCR enrichment steps were performed. A Qubit 2.0 fluorometer (Life Technologies, Carlsbad, CA, USA) was used to measure library concentration. An Agilent 2100 was used to check insert size using diluted cDNA library samples (1 ng/µL), and qRT-PCR was also performed to quantify library samples. HiSeq platform (Illumina, San Diego, CA, USA) was used for RNA-Seq (Novogene Corp. Inc., Sacramento, CA, USA).

### 4.3. De Novo Transcriptome Assembly and Functional Annotation

The raw reads were cleaned by removing reads that had adaptor contamination; more than 10 percent uncertain nucleotides or more than 50 percent of the reads constituted low-quality nucleotides (base quality is less than 5). The rest of the analysis was performed using the cleaned reads. The transcriptome assembly was performed using Trinity [75]. The transcriptome was reconstructed by forming contig assemblies, clustering those assemblies into components, constructing complete de Brujin graphs for each component, and then reconciling the de Brujin graphs to reconstruct distinct isoforms for splice transcripts. The final results were outputted in a FASTA file. Seven databases were used for functional gene annotation. These databases consisted of NR (NCBI non-redundant protein sequences), NT (NCBI nucleotide sequences), PFAM (Protein Family), KOG/COG (euKaryotic Orthologous Groups/Cluster of Orthologous Groups of proteins), Swiss-Prot, KEGG (Kyoto Encyclopedia of Genes and Genome) and GO (Gene Ontology).

### 4.4. Differential Gene Expression Analysis

The cleaned raw reads were mapped onto the assembled transcriptome. The mapping results returned a read count for each transcript. RSEM (1.2.28) was used to map reads back to the transcriptome, quantify the expression levels, and convert them into Fragments per Kilobase of transcript sequence per million base pairs sequenced (FPKM) [76]. The DESeq2 (1.26.0) R package was then used to normalize the read counts and performed a differential expression analysis based on the negative binomial distribution for its estimation model [77]. To control the false discovery rate (FDR), Benjamini and Hochberg’s method was used to calculate adjusted *p*-values. The significance threshold for the adjusted *p*-values was set to α = 0.05. Genes deemed significantly differentially expressed needed to satisfy both *p*_adj_ ≤ 0.05 and |log_2_(fold change)| ≥ 2.

Once the differentially expressed genes were gathered, Venn diagrams and a heatmap were constructed. The Venn diagrams were made using the “VennDiagram” R package, and the heatmap was constructed using the pheatmap R package (https://cran.r-project.org/web/packages/pheatmap/index.html; accessed on 12 September 2021) using the top 100 most significant genes [78].

### 4.5. Gene Ontology Annotation and Enrichment Analysis of DEGs

The GOseq (1.32.0) R package was used to perform a Gene Ontology (GO) analysis of the differentially expressed genes [79]. All three GO terminologies (biological processes, cellular component, molecular function) were considered in the GO distribution. Assuming that all genes have the same probability of being chosen within a category, the Wallenius non-central hypergeometric distribution could perform the GO enrichment test. Over-representation of the DEGs was tested with an adjusted *p*-value threshold of α = 0.05. The effect size calculation was performed by taking the *p*-value and performing a −log_10_ transformation.

### 4.6. KEGG Pathway Enrichment Analysis of DEGs

The Kyoto Encyclopedia of Genes and Genomes, or KEGG (http://www.genome.jp/kegg/; accessed on 11 September 2021), is an accumulation of databases associated with genomes and biological pathways. The hypergeometric distribution was applied to perform a KEGG enrichment test using the clusterProfiler R package [80,81]. To control the false discovery rate across multiple tests, adjusted *p*-values were computed and used when determining significance.

### 4.7. Functional Analysis and Visualization

Using the built-in “grep” R function, specific keywords were searched for in the NR descriptions of each comparison. Genes with log_2_ fold change greater than zero were colored blue, while genes with log_2_ fold change lesser than zero were colored red.

### 4.8. Transporter Analysis

Mappings to Transporter families were obtained from the Transporter Classification Database (https://tcdb.org/; accessed on 23 September 2021). Protein family (PFam) IDs were used to map the genes to their associated families and superfamilies.

### 4.9. Transcription Factor and Regulator Analyses

To perform transcription factor and regulator analyses, iTAK and HMMER (3.1) were used to perform an hmmerscan and identity families from the Transcription Factor/Regulator Database. If applicable, differentially expressed genes were mapped to their respective families and sub-families [82,83].

### 4.10. Quantitative Reverse Transcription PCR (qRT-PCR)

Thirty differentially expressed genes (clusters) were randomly selected for qRT-PCR analyses (Table S4). Phytozome portal (v13) was used for BLAST analyses for each gene (cluster) to identify the *Glycine max* gene with the best sequence homology. To identify the intron-exon boundaries of the guar gene, the genomic sequence of *G. max* gene was compared with the corresponding cluster sequence. In most clusters, a minimum of one primer of each pair was designed from two exons flanking an intron (Table S5).

A previously described method was used qRT-PCR assays [17]. For qRT-PCR assays, same RNA samples were used, which were also used for RNA Seq experiments. All RNA samples were treated with DNase I to remove any DNA contaminations according to the manufacturer’s instruction (Thermo Scientific, Waltham, MA, USA). Subsequently, the RNA samples were diluted to 5 ng/µL. All qRT-PCR assays were performed in BioRad CFX96 System using iTaqTM Universal SYBR^®^ Green One-Step Kit (Bio-Rad Laboratories, Hercules, CA, USA). Each qRT-PCR reaction was performed in a 10 µL volume containing 5 µL of 2× one-step SYBR^®^ Green Reaction mix, 10 ng total RNA, 0.75 µM each of forward and reverse primers, and 0.125 µL iScriptTM Reverse Transcriptase. The following conditions were used qRT-PCR: 50 °C for 10 min, 95 °C for 1 min, afterward 40 cycles of 95 °C denaturation for 10 s, 57 °C annealing for 30 s, and 68 °C extension for 30 s. RNA samples from three biological replicates and two technical replicates were used for all assays. Guar *Actin 11* (*Act11*), *elongation factor-1 alpha* (*EF-1a*), and *glyceraldehyde-3-phosphate dehydrogenase* (*GAPDH*) were used as reference genes in expression analyses [84].

## 5. Conclusions

The transcriptomics approach was employed to study molecular mechanisms of salinity tolerance in two contrasting guar genotypes: Matador (‘03’), which is salt tolerant, and PI 340261 (‘22’), which is salt sensitive. Thousands of DEGs identified in ‘03’ vs. ‘22’ were linked to several biological pathways, including hormonal signaling, calcium signaling, redox signaling, transcriptional regulation, microRNA biogenesis, and post-transcriptional regulation in guar. This study also revealed upregulated expression of a gene belonging to the cation/proton antiporter 1 (CPA1) family that plays a role in Na^+^ homeostasis and a gene encoding *KUP* which contributes to K^+^ homeostasis in response to salinity. It also appears that the salt-tolerant ‘03’ has a better ability to suppress the expression of genes that function as negative regulators of salinity tolerance in plants (e.gs. *GIGANTEA* and *E3 ubiquitin ligase PQT3*) than ‘22’. The reverse genetics approach can be employed to examine the biological roles of some promising genes in response to salinity. Positive functional validation data for different genes would be useful to develop salt-tolerant guar genotypes, which could be cultivated in moderately saline soils or with recycled waters of elevated salinity.

## Figures and Tables

**Table 1:** Differentially expressed genes (DEGs) identified in different comparisons.

Comparison	Groups	DEGs	Upregulated	Downregulated
Treatment vs. Control	T03L vs. C03L T03R vs. C03R T22L vs. C22L T22R vs. C22R	853 8263 1070 1006	357 1805 644 511	496 6478 426 495
Tolerant vs. Sensitive	C03L vs. C22L C03R vs. C22R T03L vs. T22L T03R vs. T22R	1049 1355 1031 1727	541 583 430 538	508 772 601 1189
Leaf vs. Root	C03L vs. C03R C22L vs. C22R T03L vs. T03R T22L vs. T22R	41,540 35,936 21,383 27,383	10,259 8978 8168 8702	31,281 26,958 13,215 18,681

**Table 2:** Salt ion composition of control and saline irrigation water treatments. Water pH = 7.4.

	EC_iw_		Ion Concentration in Mmol_c_ L^−1^							
Treatment	dS m^−1^	NO_3_^−^	SO_4_^2−^	Cl^−^	PO_4_^3−^	CO_3_H^−^	Ca^2+^	Mg^2+^	Na^+^	K^+^
Control	1.46	5.4	1.44	1.41	1.5	4.2	3.35	2.1	1.88	6.6
Saline	13.65	5.4	27.32	128.4	1.5	3.5	29.6	23	106.9	6.6

## Data Availability

Illumina HiSeq generated RNA-Seq reads are available in NCBI Sequence Read Archive (SRA) for the Bioproject: PRJNA763938 (https://www.ncbi.nlm.nih.gov/sra/PRJNA763938) (Release date: 1 February 2022). Additional datasets supporting this research are included in the paper and as Supplementary Materials.

## Supplementary Materials

The following are available online at https://www.mdpi.com/article/10.3390/plants11030291/s1, Figure S1. Length distribution of guar transcripts and unigenes. Figure S2. Functional annotation data of Guar transcriptome assembly. Figure S3. Venn diagram analysis of differentially expressed genes (DEGs). Table S1: The experimental arrangement shows leaf and root samples from two guar genotypes 03 (Matador) (salt-tolerant) and 22 (PI 340261) (salt-sensitive) subjected to control and salt treatment. Table S2: Summary of the RNA-Seq reads of 24 libraries of guar. Table S3. Expression comparison and gene ontology annotation of DEGs. Different sheets represent different comparisons. Table S4. List of guar genes (clusters) used for expression analyses by qRT-PCR Assays. Table S5. List of primers used for the qRT-PCR analyses. Table S6. Summary for gene ontology (GO) enrichment analysis of DEGs. Table S7. KEGG enrichment analysis of DEGs. Different sheets represent different comparisons. Table S8. DEGs associated with hormonal signaling. Table S9. Differentially expressed genes (DEGs) involved in calcium signaling. Table S10. Differentially expressed genes (DEGs) involved in Redox signaling. Table S11. Differentially expressed genes (DEGs) involved in transport. Table S12. Number of transporter families identified in different comparisons. Table S13. Differentially expressed genes (DEGs) encoding transcription factors/regulators. Table S14. Number of family members of transcription factors/regulators.
