# Supplementary material for: Deciphering Molecular Mechanisms Involved in Salinity Tolerance in Guar (Cyamopsis tetragonoloba (L.) Taub.) Using Transcriptome Analyses
Source: Plants (Basel). 2022 Jan 22;11(3):291. doi: 10.3390/plants11030291 (PMC8838131; doi:10.3390/plants11030291)
Supplement: Supplementary file 1 [file plants-11-00291-s001.zip › plants-1530720 (1)/Supplemental-Figsplants-1530720.pptx]

## Slide 1
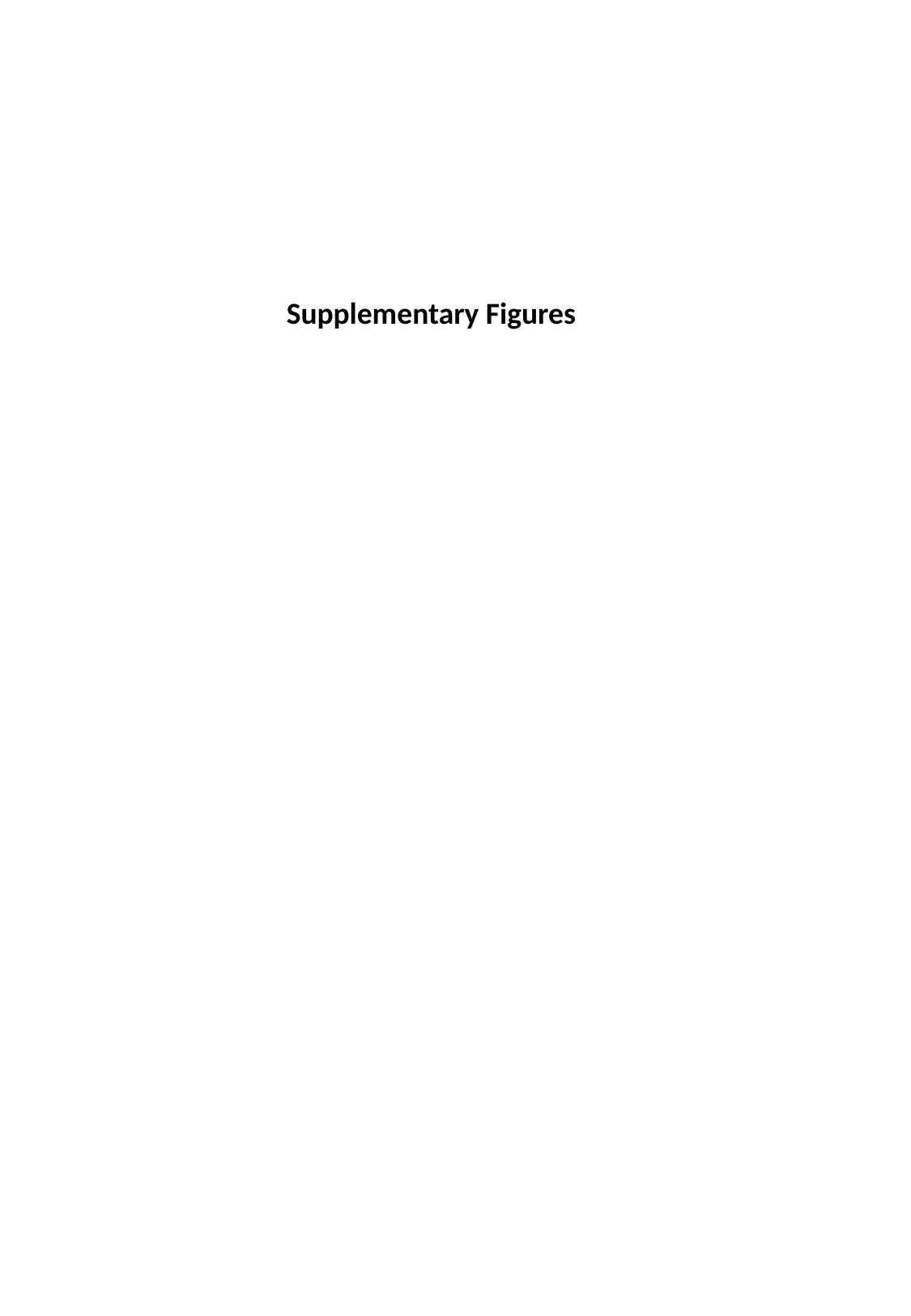

Supplementary Figures

## Slide 2
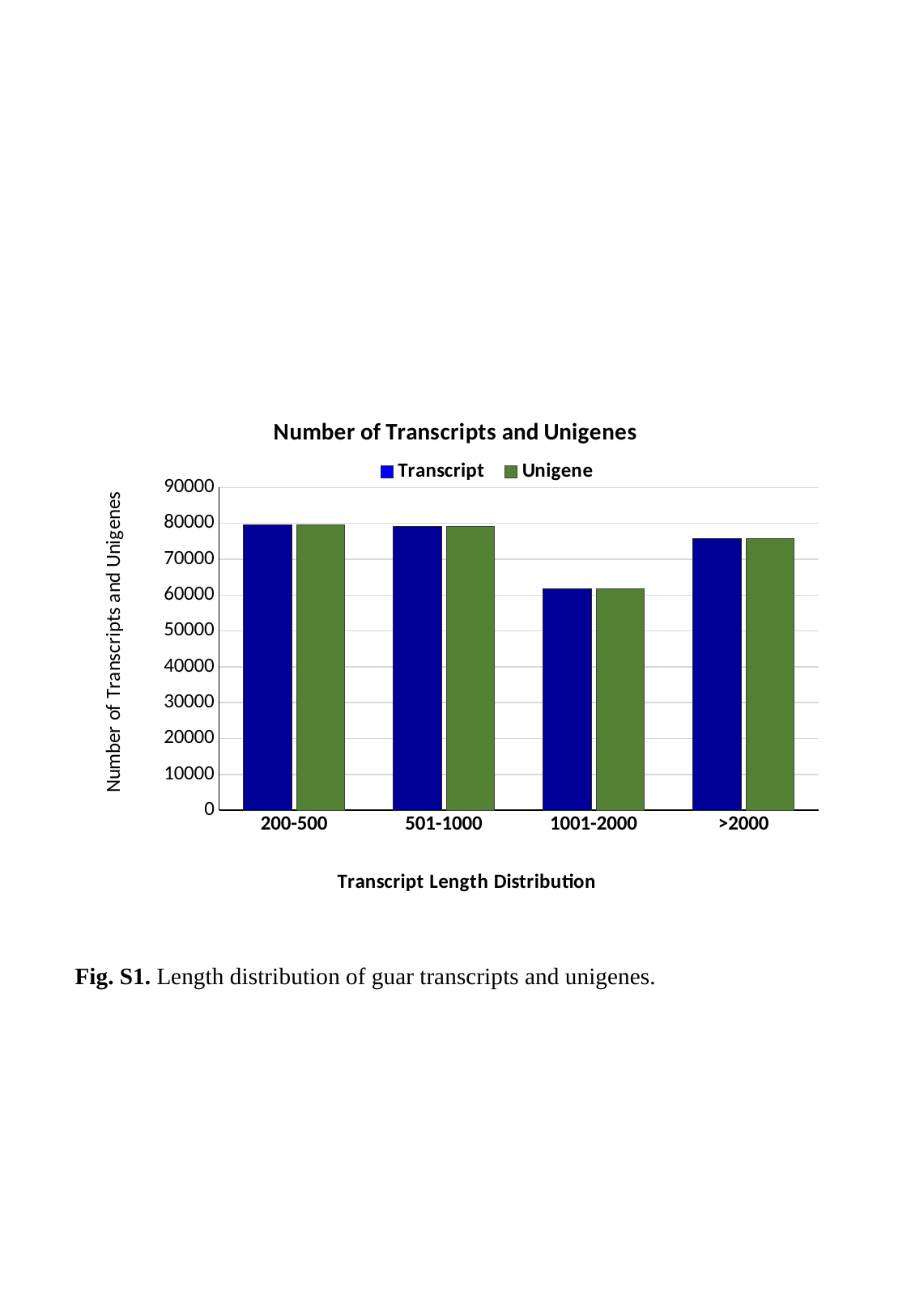

### Chart: Number of Transcripts and Unigenes
| Category | Transcript | Unigene |
|---|---|---|
| 200-500 | 79657.0 | 79607.0 |
| 501-1000 | 79165.0 | 79162.0 |
| 1001-2000 | 61650.0 | 61650.0 |
| >2000 | 75695.0 | 75695.0 |Fig. S1. Length distribution of guar transcripts and unigenes.

## Slide 3
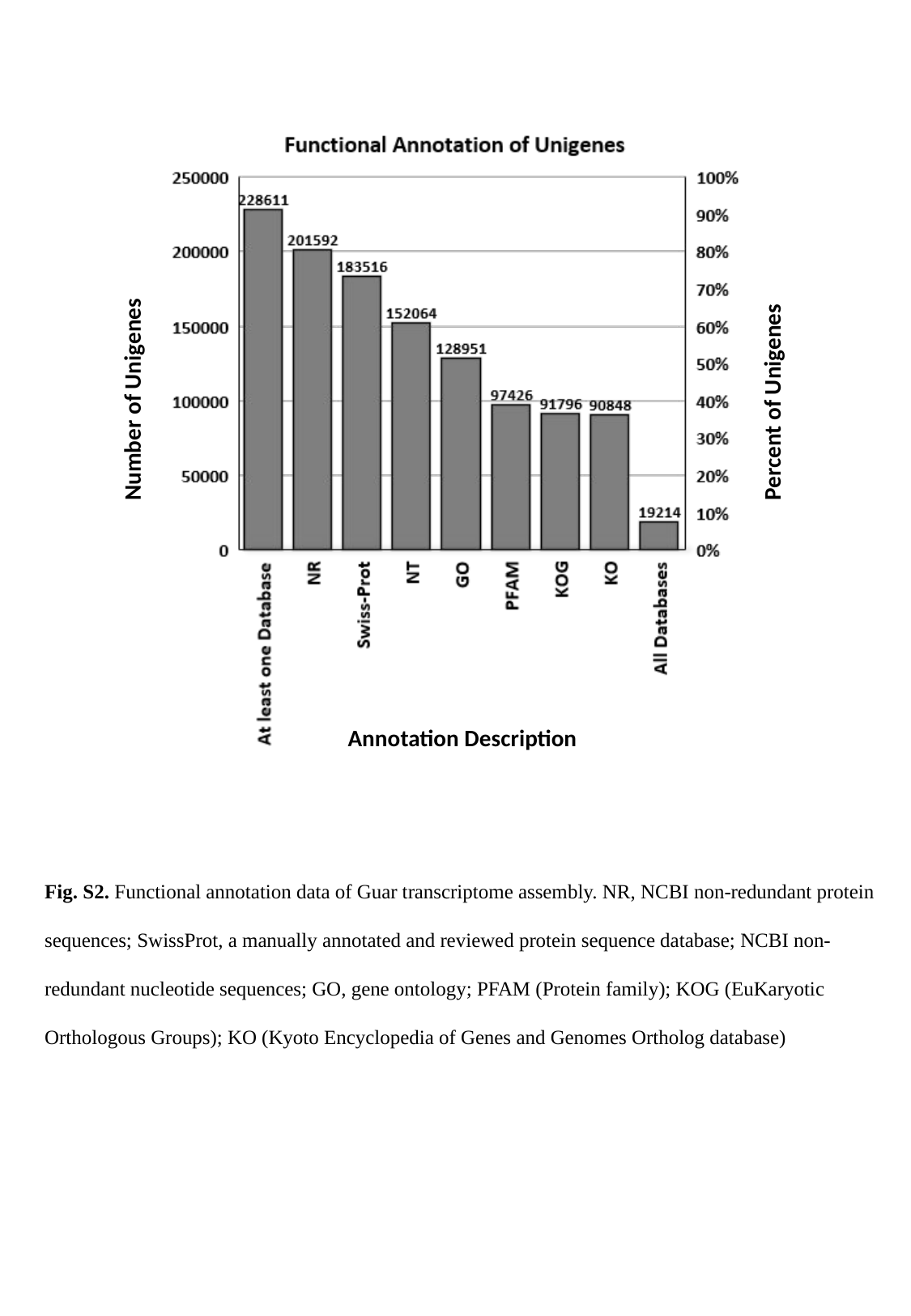

Number of Unigenes
Percent of Unigenes
Annotation Description
Fig. S2. Functional annotation data of Guar transcriptome assembly. NR, NCBI non-redundant protein sequences; SwissProt, a manually annotated and reviewed protein sequence database; NCBI non-redundant nucleotide sequences; GO, gene ontology; PFAM (Protein family); KOG (EuKaryotic Orthologous Groups); KO (Kyoto Encyclopedia of Genes and Genomes Ortholog database)

## Slide 4
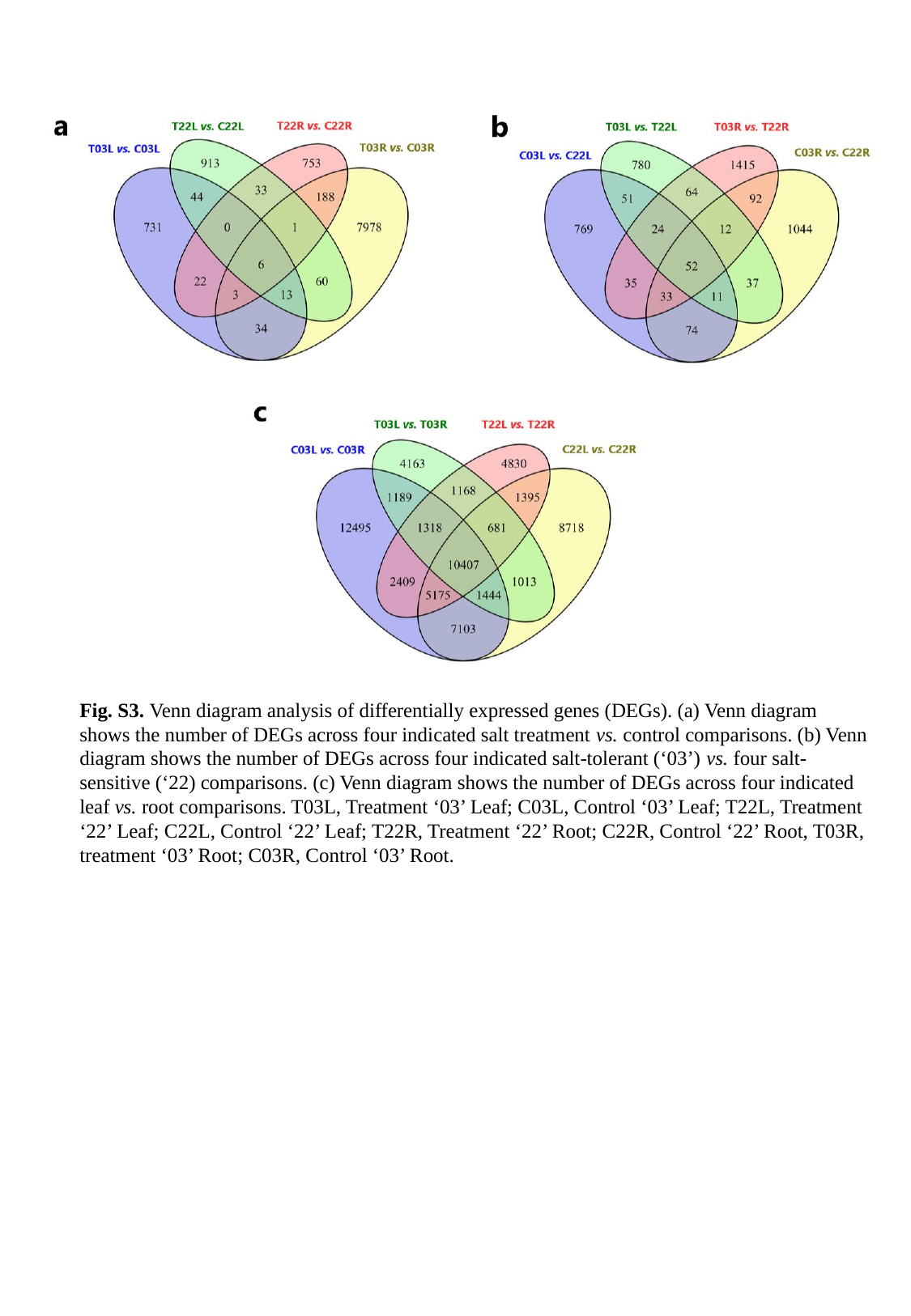

Fig. S3. Venn diagram analysis of differentially expressed genes (DEGs). (a) Venn diagram shows the number of DEGs across four indicated salt treatment vs. control comparisons. (b) Venn diagram shows the number of DEGs across four indicated salt-tolerant (‘03’) vs. four salt-sensitive (‘22) comparisons. (c) Venn diagram shows the number of DEGs across four indicated leaf vs. root comparisons. T03L, Treatment ‘03’ Leaf; C03L, Control ‘03’ Leaf; T22L, Treatment ‘22’ Leaf; C22L, Control ‘22’ Leaf; T22R, Treatment ‘22’ Root; C22R, Control ‘22’ Root, T03R, treatment ‘03’ Root; C03R, Control ‘03’ Root.
