# Supplementary material for: Deciphering Molecular Mechanisms Involved in Salinity Tolerance in Guar (Cyamopsis tetragonoloba (L.) Taub.) Using Transcriptome Analyses
Source: Plants (Basel). 2022 Jan 22;11(3):291. doi: 10.3390/plants11030291 (PMC8838131; doi:10.3390/plants11030291)
Supplement: Supplementary file 1 [file plants-11-00291-s001.zip › plants-1530720 (1)/Table_S1_11052021.pdf]

Table S1. The experimental arrangement shows leaf and root samples from two guar genotypes '03 (Matador)' (salt-tolerant) and '22 (PI 340261)' (salt-sensitive) subjected to control and salt treatment

Tissue	Replicates	Control		Salt treatment	
		**'03 (Matador)'**	**'22 (PI 340261)'**	**'03 (Matador)'**	**'22 (PI 340261)'**
**Leaf**	R1	C03L1	C22L1	T03L1	T22L1
	R2	C03L2	C22L2	T03L2	T22L2
	R3	C03L3	C22L3	T03L3	T22L3
**Root**	R1	C03R1	C22R1	T03R1	T22R1
	R2	C03R2	C22R2	T03R2	T22R2
	R3	C03R3	C22R3	T03R3	T223
